# Field study on routine procedures for navel care in neonatal calves on dairy farms in Eastern Germany

**DOI:** 10.1371/journal.pone.0329326

**Published:** 2025-07-30

**Authors:** Kim Kristin Meier, Kerstin-Elisabeth Müller, Roswitha Merle, Heidi Arndt, Linda Dachrodt, Martina Hoedemaker, Laura Kellermann, Gabriela Knubben-Schweizer, Maria Volkmann, Annegret Stock

**Affiliations:** 1 Farm Animal Clinic, Division for Ruminants and Camelids, Unit for Internal Medicine and Surgery, School of Veterinary Medicine, Freie Universität Berlin, Berlin, Germany; 2 Institute of Veterinary Epidemiology and Biostatistics, School of Veterinary Medicine, Freie Universität Berlin, Berlin, Germany; 3 Clinic for Cattle, University of Veterinary Medicine Hannover, Foundation, Hannover, Germany; 4 Department of Behavioral Physiology of Livestock, Institute of Animal Science, University of Hohenheim, Stuttgart, Germany; 5 Clinic for Ruminants with Ambulatory and Herd Health Services, Centre for Clinical Veterinary Medicine, Ludwig-Maximilians-Universität Munich, Oberschleissheim, Germany; Michigan State University, UNITED STATES OF AMERICA

## Abstract

Clean conditions and prophylactic measures around calving are essential for the health and welfare of calves. Therefore, the objective of this study was to evaluate the association of different navel care (NC) practices on the occurrence of omphalitis in neonatal dairy calves. Between December 2016 and July 2019, 196 dairy farms in Eastern Germany were visited once within a large-scale cross-sectional study. 1,967 calves aged five to 21 days were clinically examined, including palpation of the external umbilicus for inflammation signs. Furthermore, information on animal health and farm management, including the implementation of NC, was obtained through interviews with the farm or herd manager. Causal diagrams were drawn, containing variables considering NC (practice of NC, method of application, preparation applied, frequency of NC, time of first NC, wearing gloves during NC) as influence variables, omphalitis as target variable, and all potential confounders to perform multivariable statistical analyses at animal level. Over one-fourth of all calves examined showed omphalitis signs (n = 525 calves, 26.7%). The odds of omphalitis tended to increase (OR = 2.3) if no NC was performed compared to regular NC. Almost half of all other variables analysed seemed relevant for the occurrence of omphalitis. Administering the preparation into the umbilical cord reduced the odds of omphalitis by 62% compared to no NC. Repeated applications tended to decrease the odds of omphalitis by 44% compared to single applications. Furthermore, wearing gloves during NC tended to increase the odds of omphalitis by 30% compared to not wearing gloves. Neither the preparation applied, the method of application, nor the timing of NC had an impact on the omphalitis occurrence. Considering these results, different NC practices influence the odds of omphalitis in neonatal dairy calves. Nevertheless, further investigations are necessary regarding the application procedure of NC during the daily farm routine.

## Introduction

Neonatal morbidity and mortality have become highly prioritized welfare issues in dairy farming [1–3]. Therefore, prophylactic measures based on scientific evidence, as well as clean calving areas, need to be implemented in the process of calf rearing. Perrot et al. demonstrated that umbilical infections are one of the most common disorders in calves, which are promoted by unhygienic bedding of the calving area [4]. Studies concerning the umbilical health of calves report huge differences in the prevalence of omphalitis. The reported prevalence of omphalitis from clinical examinations ranges from 3.8% to 28.7% in dairy calves [5,6] and from 10.3% to 32.3% in beef calves [4,7]. In addition, the results of necropsies performed on 187 Holstein-Friesian bull calves aged between seven and fifteen days revealed umbilical infections in more than one-third of these calves (34.2%) [8].

The most common disorder of the umbilicus in neonatal calves is acute omphalitis [9], characterized by increased circumferential swelling of the cutaneous part of the umbilicus, increased heat and a pain reaction on palpation, delayed drying of the umbilical cord and, in some cases, purulent discharge [8,10]. Ascending infections of the intra-abdominal umbilical structures may occur without any externally visible signs of inflammation [11]. Therefore, they can easily be missed by the farmer. The latter infections, however, carry the risk of abscess formation in the liver, urinary bladder infections, or the spread of pathogens via the bloodstream, leading to septicemia and/or joint colonization with subsequent polyarthritis [11].

The disparity between the number of dairy calves exhibiting signs of umbilical inflammation during clinical examinations on a single farm visit and the farm records of treatments for omphalitis in the twelve months preceding this visit was previously reported by our group. Although a median of 25.0% of calves showed signs of umbilical inflammation (swelling with or without warmth, reddening, or pain reaction on palpation), only 4.5% of calves were treated in the previous twelve months [12]. This difference highlights the problem of underestimation of the number of umbilical infections on dairy farms due to a lack of detection of affected calves.

Umbilical infections predominantly affect calves in the first three weeks of life, with a median age of 18 days [5]. Colonization of the umbilical structures by opportunistic Gram-positive and Gram-negative bacteria, including those from faeces, skin, mucosa, and the environment, in the period around birth has been shown to contribute to umbilical infections [13,14]. Since the umbilical cord remnant (UCR) (amniotic remnant) was demonstrated to allow pathogens to invade the umbilicus [8], its quick drying off should be pursued to close the entry road and, therefore, prevent umbilical infections [15]. To promote umbilical involution, accelerate drying off, and reduce the bacterial load at the UCR, navel care (NC) at birth is widely recommended and practiced [16]. Among the most commonly implemented NC practices is the topical application of antiseptics such as iodine or chlorhexidine (CHX), applied by spray or dip, a practice that has been in use for nearly two decades [17]. Due to the scarcity of controlled clinical studies on the topic of NC, however, the need and efficacy of NC are still under discussion.

The antiseptics mostly recommended and used include CHX and various iodine compounds [16,18,19]. Iodine is bactericidal, sporicidal, and virucidal [20], with a rapid onset of effect even in low concentrations [21], ensuring significant bacterial reduction [22]. As iodine is neutralized by organic material, repeated application is necessary to provide adequate disinfection [20]. Potential adverse side effects provoked by iodine compounds in calves and farm workers, such as skin irritations and staining [20,21], may limit the use of iodine for prophylactic NC [23]. The use of iodine for NC in infants was linked to iodine overload, hypothyroidism, and thyroid blockade [24]. In Germany, different concentrations and formulations of iodine are authorized for NC in calves (e.g., 10% povidone-iodine solution (Vet-Sept Lösung 10% [25]), 5% povidone-iodine spray (Vet-Sept Spray [26]), 2.5% iodine and 2.5% potassium iodide in an alcoholic solution (Alkoholische Jodlösung WDT [27])). In other countries, however, strict restrictions were implemented for the use of strong iodine (greater than 2.2% iodine, e.g., 7% iodine) to prevent the use of iodine crystals for the illegal production of methamphetamine in clandestine drug laboratories [28]. Povidone-iodine, however, what is frequently used for NC in calves, is a mixture of povidone and hydrogen iodine [29]. Therefore, the concentration of iodine itself is lower compared to formulations with pure iodine (e.g., 7% iodine). A 10% povidone-iodine solution, for example, contains approximately 1% available iodine and iodide [30].

Many studies in animals and infants have shown that CHX delays umbilical cord drying and detachment [15,31–33] while significantly reducing bacterial colonization [32,33], umbilical infections, and the mortality rate [34–37]. Furthermore, CHX is effective against a broad spectrum of bacteria with few side effects [21]. Therefore, this makes it a suitable umbilical antiseptic with high skin compatibility [23]. In Germany, however, iodine is the only authorized antiseptic for NC in calves.

The aim of the present cross-sectional study was to gain insight into the routines on dairy farms in Eastern Germany concerning different navel care practices and to evaluate these measures in light of the results of clinical examinations of neonatal calves conducted during farm visits on a single occasion.

## Materials and methods

The data used in the present study originates from a cross-sectional field study performed on dairy farms located in three different regions (North, East, and South) of Germany from December 2016 to July 2019 (PraeRi) [38,39]. The study aimed to assess the status of husbandry conditions, hygiene, feeding, animal health, and management practices. This paper evaluates data from a survey conducted in Eastern Germany. No ethical or animal experiment approvals were necessary due to the legal regulations at that time in Germany. All participants provided written consent to participate in the study and were informed that the data would be analysed anonymously and that they could withdraw from participation at any time point without any consequences.

Farmers were recruited by postcard from October 2016 until June 2019. If farmers were interested in participation, the study veterinarians contacted them by phone. The study was explained once again, and information regarding the upcoming farm visit was requested. The detailed sample size calculation and the recruitment process of the farms were described by Merle et al. [39]. Based on the number of milking and dry cows retrieved from the National Traceability and Information System for Animals (“Herkunftssicherungs- und Informationssystem für Tiere”, HIT) farms were categorized in small, medium, and large (Table 1).

**Table 1:** Categorization of the farm sizes in the eastern region based on the number of milking and dry cows retrieved from HIT^1^.

Farm size	Small	Medium	Large
**Number of milking and dry cows**	1–160	161–373	≥ 374

^1^ HIT: National Traceability and Information System for Animals (“Herkunftssicherungs- und Informationssystem für Tiere”)

Each farm was visited once by trained veterinarians. The study veterinarians conducted interviews with the farm or herd managers and performed clinical examinations of the calves according to a standardized protocol. Once a year, training sessions were implemented to ensure inter-observer reliability among the different study veterinarians. A detailed description of the implementation is described in Dachrodt et al. [40].

### Interview with the farm or herd manager

The questionnaire contained questions about farm management, husbandry, feeding, biosecurity, and animal health. For this analysis, 13 questions were included. Table 2 gives an overview of the questions included. All questions and possible answers are listed in the S1 Table in the supporting information.

**Table 2:** Information retrieved and evaluated from questionnaires and HIT^1^.

Variable	Source	Farm or animal level
**Farm management**		
Farming type	Questionnaire	Farm level
Farm size (number of lactating and dry cows)	HIT	Farm level
Existence of calf care workers	Questionnaire	Farm level
Number of full- and part-time employees^2^	Questionnaire	Farm level
**Calving management**		
Main calving pen	Questionnaire	Farm level
General time calves spend with the dam after birth	Questionnaire	Farm level
**Standard operation protocols (SOP)**		
SOP for health checks in calves	Questionnaire	Farm level
**Navel care (NC)** ^*^		
Practice of navel care	Questionnaire	Animal level
Method of application	Questionnaire	Animal level
Administering the preparation into the umbilical cord	Questionnaire	Animal level
Preparation or product applied	Questionnaire	Animal level
Frequency	Questionnaire	Animal level
Time between birth and NC	Questionnaire	Animal level
Wearing gloves during NC	Questionnaire	Animal level
**Calf related variables**		
Sex	HIT	Animal level
Birth season	HIT	Animal level
Breed	HIT	Animal level

^1^ National Traceability and Information System for Animals (“Herkunftssicherungs- und Informationssystem für Tiere”)

^2^ Basis for calculation of the cows per employee

* All 7 variables used for confounder-adjusted analyses

To evaluate the effects of the human resources involved in the care for dairy cows and calves on calf health, the numbers of cows cared for by a single farm worker were calculated as follows:


$$\text{Cows per employee}\frac{\text{ = mean number of cows on the farm in the past 12 months}}{\text{number of full-time employees + number of part-time employees*0.5}}$$

The whole questionnaire is available on www.praeri.de (German) or can be requested from the author (German or English).

### Clinical examination of calves

On each farm, a representative sample of calves was clinically examined during a single farm visit based on a sample size calculation performed in advance of all farm visits. The sample size was calculated based on the number of pre-weaned calves on the farm on the day of the farm visit (aged maximum six months) (Table 3) [40]. If the number of calves on the farm exceeded the required sample size, the calves were evenly distributed among the different age and housing groups. Thus, the same percentage of calves was examined in each group (e.g., 80% of all calves in a single housing, 80% of all calves in group 1, and 80% of all calves in group 2). All calves were chosen in each group by chance. For example, on a large farm with 100 calves, 73 calves required examination (Table 3). Therefore, in this example, 3 out of 4 calves were examined. Each calf was caught and marked. Every fourth calf, however, was not clinically examined. Findings were recorded on a protocol sheet.

**Table 3:** Number of calves examined based on the number of pre-weaned calves present on the day of the farm visit (aged 6 months at maximum).

Number of pre-weaned calves	Number of calves examined
1–40	all calves
41–73	40
≥ 74	73

Sample calculation based on expected prevalence*: 40%; confidence level: 95%; power: 80%; precision: +/- 10%

* The sample size was calculated for the highest expected prevalence of any disorder (lameness) and not for the expected prevalence of omphalitis.

The examination of the umbilicus included inspection of the external umbilicus, followed by palpation, focusing on signs of inflammation (swelling with or without hyperthermia, reddening, and/or a pain response on palpation).

### Data analysis

For the statistical analyses, a hypotheses-based approach was used. Before the analyses were conducted, hypotheses were formulated, including the occurrence of omphalitis as the outcome variable and all possible influence variables identified through literature research, expert knowledge, and assumed influence. Solely variables that were evaluated during the study and were potentially modifiable by the farm manager were included. For each variable, descriptive analyses were performed at animal or farm level using SAS 9.4 (SAS Institute, Cary, North Carolina, USA). If a continuous variable was not normally distributed, it was categorized, or the logarithm of the variable to the base 10 was used to achieve a better model fit. If one category in a categorical outcome included less than 5% of observations, the categories were revised.

For all variables, univariable mixed logistic regression models were performed to examine the association between these variables and the occurrence of omphalitis at animal level. The hypotheses regarding NC that were included in this analysis are listed in the S2 Table in the supporting information.

Based on the hypotheses formulated, a large overview causal directed acyclic graph (DAG) (http://www.dagitty.net/) was developed, with the occurrence of omphalitis as the target variable and all possible influence variables evaluated during the farm visits. Arrows were drawn between the variables to demonstrate their associations. Based on the overview DAG, seven individual DAGs were created, each incorporating the respective navel care variable as an influence factor (practice of NC, method of application, administering the preparation into the umbilical cord, preparation applied, frequency of NC, time between birth and NC, and wearing gloves during NC). All variables that showed neither an association with the influence nor the target variable were excluded from the DAG. All variables that were identified by the program DAGitty as confounding variables were included in the multivariable model [41]. The causal diagrams for each variable are shown in the supporting information (S1–S7 Fig). The confounder variables for each analysis are listed in the footnotes of the respective model.

Based on these diagrams, multivariable mixed logistic regression models were performed with the occurrence of omphalitis as the dependent variable, the influence variable of interest, and all confounder variables as independent variables, along with the farm as a random factor. Farm size and farming type were included as general confounder variables in all models. The −2 Res Log Pseudo-Likelihood (−2LL) was used to assess the goodness-of-fit of each model for the univariable and multivariable analyses. The −2LL values are shown in the footnotes in Table 5.

**Table 4:** Omphalitis prevalence and description of the study population of calves aged between 5 and 21 days of life from 196 dairy farms in Eastern Germany.

Variable	% (n)
**OMPHALITIS PREVALENCE**	
**Individual animal level**	26.7 (525)
**Farm level**	
Median per farm	25.0 (196)
IQR	11.1–36.4
**STUDY POPULATION (farm level)**	
**Farming type**	
Conventional	92.9 (182)
Organic or in conversion to become organic	7.1 (14)
**Farm size (number of lactating and dry cows)**	
Median	172.5
IQR	141–298
**Cows per employee**	
Median	31.0
IQR	22.5–40.6
**Specialized calf care workers**	
Yes	60.2 (118)
No	39.8 (78)
**STUDY POPULATION (animal level)**	
**Sex**	
Female	63.1 (1,241)
Male	36.9 (726)
**Breed**	
Holstein^1^	90.6 (1,779)
Simmental	0.3 (6)
Crossbreed between dairy and beef/dual purpose breed with emphasis on beef^2^	6.4 (125)
Other dairy breeds and their crossbreeds^3^	2.8 (54)

^1^ Holstein Friesian and Red Holstein.

^2^ Crossbreed between dairy and beef breed or two beef breeds, Pinzgauer cattle, other.

^3^ Brown Swiss, Jersey, German black pied cattle, German red pied cattle, Angler, crossbreed between 2 dairy breeds.

IQR: Interquartile Range.

**Table 5:** Description, univariable, and confounder-adjusted analyses of the influence of different navel care practices on the occurrence of omphalitis in calves from 196 dairy farms in Eastern Germany at animal level.

Univariable analysis								Confounder adjusted model				
Variable	Number of farms (%)	Number of calves (%)	Crude estimate	OR	95% CI		p-value	Adjusted estimate	OR	95% CI		p-value
**Practice of navel care (NC)** ^1^ ^,^ ^2^							**0.514**					**0.397**
Always (> 90% of the calvings)	107 (54.6)	1,290 (65.6)	Reference	.	.	.	.	Reference	.	.	.	.
Infrequently	30 (15.3)	215 (10.9)	0.073	1.1	0.7	1.7	0.738	0.203	1.2	0.8	2.0	0.397
Never	59 (30.1)	462 (23.5)	0.188	1.2	0.9	1.7	0.250	0.831	2.3	Not est.	Not est.	0.056
**Method of application** ^1^ ^,^ ^3^							**0.672**					**0.473**
Spraying	80 (58.8)	853 (57.3)	Reference	.	.	.	.	Reference	.	.	.	.
Dipping	29 (21.3)	341 (22.9)	−0.213	0.81	0.5	1.3	0.332	−0.346	0.71	0.4	1.2	0.178
Pouring on	22 (16.2)	248 (16.6)	0.061	1.1	0.7	1.7	0.796	−0.06	0.94	0.5	1.6	0.829
Painting/spotting or combinations	5 (3.7)	48 (3.2)	−0.328	0.72	0.3	2.0	0.525	−0.463	0.63	0.2	1.8	0.391
**Administering the preparation into the umbilical cord** ^1^ ^,^ ^4^							**0.003**					**0.001**
No navel care	59 (30.3)	462 (23.7)	Reference	.	.	.	.	Reference	.	.	.	.
Yes	21 (10.8)	273 (13.4)	−0.82	0.44	0.3	0.7	0.001	−0.981	0.38	Not est.	Not est.	0.023
No	115 (59.0)	1217 (62.4)	−0.057	0.95	0.7	1.3	0.718	−0.109	0.9	Not est.	Not est.	0.775
**Preparation or product applied** ^5^							**0.839**					**0.987**
No navel care	59 (30.3)	462 (23.7)	Reference	.	.	.	.	Reference	.	.	.	.
Iodine	86 (44.1)	964 (49.4)	−0.142	0.87	0.6	1.2	0.378	−0.009	1.0	Not est.	Not est.	0.971
Oxy-/chlortetracycline, partially with other interventions	42 (21.5)	416 (21.3)	−0.104	0.90	0.6	1.3	0.598	0.010	1.0	Not est.	Not est.	0.971
Alcohol, chlorhexidine, or other	8 (4.1)	110 (5.6)	−0.161	0.85	0.5	1.6	0.593	−0.038	0.96	Not est.	Not est.	0.914
**Frequency of NC** ^1^ ^,^ ^6^							**0.057**					**0.065**
Single application	123 (90.4)	1341 (90.0)	Reference	.	.	.	.	Reference	.	.	.	.
Repeatedly	13 (9.6)	149 (10.0)	−0.604	0.55	0.3	1.0	0.057	−0.582	0.56	0.3	1.0	0.065
**Time between birth and NC** ^1^ ^,^ ^7^							**0.721**					**0.499**
Immediately	120 (88.2)	1376 (92.4)	Reference	.	.	.	.	Reference	.	.	.	.
Later	16 (11.8)	114 (7.7)	−0.108	0.90	0.5	1.6	0.721	−0.218	0.80	0.4	1.5	0.499
**Wearing gloves during NC** ^1^ ^,^ ^8^							**0.171**					**0.092**
No	84 (61.8)	868 (58.3)	Reference	.	.	.	.	Reference	.	.	.	.
Yes	52 (38.2)	622 (41.7)	0.241	1.3	0.9	1.8	0.171	0.293	1.3	1.0	1.9	0.092

^1^ Adjusted for farm size (log), farming type, existence of calf care workers, cows per employee, SOP for prophylactic measures for calves

^2^ Adjusted for calving pen (usual husbandry, single pen, group pen, combined pen for calving and diseased cows, pasture), general time calves spend with dam after birth, birth season, sex of the calf, method of application, time between birth and NC, preparation or product applied for NC, administering the preparation into the umbilical cord, frequency of NC; −2LL: univariable: 8835.1; −2LL multivariable: 9005.3

^3^ Adjusted for general time calves spend with dam after birth, preparation or product applied for NC, time between birth and NC, practice of navel care; −2LL univariable: 6341.3; −2LL multivariable: 6782.0

^4^ Adjusted for calving pen (usual husbandry, single pen, group pen, combined pen for calving and diseased cows, pasture), general time calves spend with dam after birth, birth season, method of application, time between birth and NC, preparation or product applied for NC, practice of navel care; −2LL univariable: 8788.5; −2LL multivariable: 6800.9

^5^ Adjusted for SOP for prophylactic measures for calves, practice of navel care, farming type, farm size (log); −2LL univariable: 9759.7; −2LL multivariable: 9775.6

^6^ Adjusted for general time calves spend with dam after birth, preparation or product applied for NC, time between birth and NC, method of application, administering the preparation into the umbilical cord, sex of the calf, birth season of the calf, practice of navel care; −2LL univariable: 6738.0; −2LL multivariable: 6846.0

^7^ Adjusted for general time calves spend with dam after birth, preparation or product applied for NC, practice of navel care; −2LL univariable: 6733.6; −2LL multivariable: 6771.6

^8^ Adjusted for preparation or product applied for NC, method of application, administering the preparation into the umbilical cord, frequency of NC, practice of navel care; −2LL univariable 6736.0; −2LL multivariable: 6808.5

OR: odds ratio

95% CI: 95% confidence interval

SOP: standard operation protocol

Log: logarithmic scale

Bold numbers: global p-value for the respective model

Not est.: value not estimable

-2LL: −2 Res Log Pseudo-Likelihood

Odds ratios (OR), including 95% confidence intervals (95% CI), were calculated for the respective influence variable.

As the focus of this analysis was to determine the effect of NC practices on the development of omphalitis, the study population was limited. The age distribution of calves with signs of omphalitis showed a peak during the first days of life, which declined after the first four days (predominantly a slight swelling of the umbilicus without further signs of inflammation). In the first days of life, however, a swollen umbilicus may be physiological due to individual differences influenced by birth weight or sex [42] or may have other causes than omphalitis (e.g., haematoma). Therefore, a correct diagnosis of omphalitis by palpation alone is difficult. As most umbilical infections occur in the first three weeks of life [5], the analysis only contained data from calves aged between five and 21 days.

## Results

### Study population

In total, 1,967 calves aged between five and 21 days from 196 farms in Eastern Germany applying different navel care (NC) practices were included in this analysis. The main breeds included were Holstein Friesian and Red Holstein (90.6%).

Overall, 525 of 1,967 calves examined (26.7%) showed signs of omphalitis. The majority of farms were run conventionally (n = 182, 92.9%), while 7.1% of the farms (n = 14) were organic or in the process of conversion to organic. The study population is described in Table 4.

### Navel care

The results of the descriptive, univariable, and confounder-adjusted analyses are shown in Table 5.

In East Germany, 252 farms were visited. Only farms with calves examined between five and 21 days of age were considered for this analysis. Of these farms, 196 farms with complete questionnaires regarding NC practices were included in the study. Half of all farmers (n = 107, 54.6%) stated they always performed NC in their neonatal calves, while 15.3% did so infrequently. “Infrequently” was not further defined. The classification was used if NC was performed in less than 90% of the calvings. For some farms, it meant performing NC in specific calves (e.g., only female calves), while for other farms, it was limited to certain situations (e.g., only during summer months). The remaining 30.1% did not perform NC at all.

Most farms applied a spray for NC (58.8%), followed by dipping of the navel (21.3%). Some farms additionally administered the preparation into the umbilical cord (10.8%).

The most commonly applied products contained iodine compounds (44.1%), followed by preparations containing oxy- or chlortetracycline, partially in combination with other compounds (21.5%).

On most farms, the navel was treated once (90.4%). Most farms (88.2%) performed NC immediately after birth. On one-third of all farms, workers were instructed to wear gloves during NC (38.2%).

If no NC was performed, the odds of omphalitis tended to be higher compared to always performing NC (OR = 2.3, p = 0.056). Some of the confounder-adjusted analyses showed an association or tendency between the way of NC and the occurrence of omphalitis, as well. Comparing administering the preparation into the umbilical cord and no NC, the odds of omphalitis were reduced by administering the preparation into the umbilical cord (OR = 0.38, p = 0.001). Performing NC without administering the preparation into the umbilical cord compared to no NC, however, had no association with the odds of omphalitis in this study. Nevertheless, a repeated application of NC showed a tendency to reduce the odds of omphalitis compared to a single application (OR = 0.56, 95% confidence interval = 0.3–1.0, p = 0.07). After adding all possible confounder variables to the model, calves from farms where workers wore gloves during NC tended to have higher odds of omphalitis than calves from farms where workers did not wear gloves during NC (OR = 1.3, 95% CI = 0.95–1.9, p = 0.092). The product applied, the method of application, and the time of NC showed no associations with the occurrence of omphalitis.

## Discussion

In this cross-sectional study, different navel care (NC) practices in neonatal calves on dairy farms in Eastern Germany were investigated, and associations between the odds of omphalitis and the respective NC practice were observed. More than a quarter of all calves examined showed signs of omphalitis, which is in line with reports from other authors [4,5]. Umbilical infections were shown to affect the health and well-being of calves, increase the risk of calf mortality [43,44] and morbidity [45,46], and decrease weight gain [47]. Our analyses showed that performing no NC tended to increase the odds of being affected by omphalitis compared to calves undergoing a regular NC. Administering the preparation into the umbilical cord, however, reduced the odds of omphalitis in our study population. A repeated NC and not wearing gloves during NC seemed to decrease the odds of omphalitis, as well.

The European Food Safety Authority (EFSA) (2012) recommends repeated NC to prevent umbilical infections [48]. Literature, however, shows that in practice, only a small percentage of farms perform NC in newborn calves as a routine procedure (35% to more than 90% of farms, depending on the region) [18,38,49]. This disparity between recommendation and reality may be due to a lack of agreement on the prophylactic effect of NC, as well as the costs associated with NC (labor and product).

Many studies describe a positive effect of NC on calf health in general. Some authors consider NC to be important to prevent umbilical infections [50], and reduce morbidity [51,52] and mortality in calves [45,48,53]. They also see a risk to animal welfare and health if NC is omitted [53,54]. Other studies, however, found no impact on disease incidence, daily weight gain, mortality, or umbilical infections [12,55] or even demonstrated an adverse effect of NC on the risk of bovine respiratory diseases [56]. Abscess formation may be introduced iatrogenically due to the local application of antiseptics in high concentrations (e.g., 7% iodine solution) as well [57]. In our study, no statistically significant association was found between the occurrence of omphalitis and the practice of NC. A tendency, however, could be shown. The odds of being affected by omphalitis were 2.3 times higher when no NC was performed compared to regular NC.

In this study, the predominant preparation applied on most farms was an antiseptic based on iodine, which is in line with observations made by other authors [18,19,58]. Most studies comparing different antiseptics for NC have used 7% iodine in one group, which is considered the gold standard for local NC. However, conclusive studies on this topic are lacking [59].

One study on neonatal foals, however, showed that the usage of iodine led to a higher number of umbilical infections compared to topical application of alcohol [60]. Furthermore, it increased the time until umbilical cord remnant (UCR) drying off [61], even though it achieved a significant reduction of the bacterial load [23]. Potential skin irritations caused by iodine compounds in both animals and farm workers also limit the use of iodine [20,23].

The second most commonly applied preparations were the broad-spectrum antibiotics oxy- or chlortetracycline, partially in combination with other interventions (e.g., iodine). Both pharmaceuticals are primarily used in a spray formulation with the indication “treatment of superficial wounds of the skin contaminated with bacteria that are tetracycline sensitive” and are not authorized for NC. The usage of antibiotics in NC does not represent a measure of disinfection or accelerate the drying off of the UCR, but is a treatment with a pharmaceutical. The application on atrophying structures, such as the umbilical cord, bears the risk of developing antimicrobial resistance [62] in calves as well as in farm workers through direct or indirect contact [63,64]. Therefore, in view of prudent drug use, topically applied antibiotics should not be used in NC.

In pediatrics, the application of chlorhexidine (CHX) is recommended for NC [65]. On farms in our study, CHX was used only rarely due to the lack of authorized antiseptics for NC in Germany at the time.

In general, most studies comparing the effects of different navel antiseptics on umbilical health in dairy calves have limitations in their study design. On the one hand, they lack control groups [10,16,66], and on the other hand, they include short observation periods (e.g., 48 hours) [16,61]. Therefore, it is difficult to conclude whether NC has a preventive effect compared to no NC [59]. Only one study included a control group and reported fewer umbilical infections in calves whose navels were disinfected with 7% iodine, 0.5–2.0% iodine, or Navel Guard (commercially available dip with antimicrobial properties and isopropyl alcohol) compared to no disinfection [50]. The comparison of other antiseptics with each other (e.g., 7% iodine, 4% CHX, 0.1% chlorine, 10% trisodium citrate, Navel Guard), however, did not show significant differences in reduction of umbilical infections [16,66], healing time of the UCR [16] or involution of the umbilical structures (comparison of 10% iodine and oxytetracycline hydrochloride) [67]. In this study, no differences in the occurrence of omphalitis were observed in calves in relation to the product applied. Therefore, it is not possible to make a recommendation for a specific product based on the recent literature and our results.

Different NC practices, regardless of the preparation applied, are rarely evaluated. The recommendation for implementing NC in calves involves repeated applications shortly after birth [48]. The umbilical cord should be dipped without wetting the surrounding tissue (to avoid skin irritation). For each calf, an individual dipping cup should be used [68]. The product used must be discarded after use to prevent cross-contamination [29]. A spray antiseptic, however, can be used to apply a clean solution on a large number of calves [68]. Due to the ease of application, most farms in this study used spray instead of a dip, whereas in other studies, the distribution is balanced [69]. Nevertheless, the spray application bears the risk of missing areas of the umbilicus, thereby reducing its effectiveness [29].

Most farms, however, disinfect the navel only once immediately after birth. This finding agrees with observations on other farms [69,70]. Repeated applications appeared to be relevant in reducing the odds of omphalitis in our study population, which is in line with another recent study [18]. A pediatric review described a positive effect of repeated applications of an antiseptic, as well, due to a reduction in the number of omphalitis cases and the bacterial load [31]. Excessive or incorrect disinfection, however, may lead to skin irritation, which may prolong UCR drying, impair the healing process, and potentially result in umbilical infections [71].

On farms where the preparations were administered into the UCR, the odds of omphalitis were lower compared to calves from farms without any NC. Only preparations authorized to be administered into the UCR should be used to avoid the entrance of potentially irritating formulations into the abdomen. In Germany, for instance, 10% PVP-iodine is authorized for administering into the UCR, even though cytotoxic effects of PVP-iodine have been described [72]. Nevertheless, its cytotoxicity seems to be lower compared to other antiseptics (e.g., CHX) [73,74]. The positive effect of administering the preparation into the UCR we found in our study might be due to the preparation applied itself or other management or farm factors that were not evaluated in this analysis (e.g., colostrum management and hygienic conditions of the calving pen). In farms where employees administered the preparation into the UCR, they may have been more conscientious about umbilical health in general. Furthermore, the way of administering the preparation into the UCR was not evaluated. We assumed that in cases where the antiseptic was administered into the distal part of the UCR but did not pass the abdominal wall, the effect of promoting UCR drying may have outweighed the adverse effects, as the connection between the environment and abdomen closes more quickly.

The impact of the frequency of NC and administering the antiseptic into the umbilical cord may be associated with the antiseptic applied rather than the method of application per se. Depending on the preparation applied, the manufacturer provides different instructions for use. In Germany, for example, various products containing different iodine concentrations and formulations with varying instructions for use (spray vs. administering into the UCR vs. pouring on) are authorized for NC. Some preparations are advised to be administered into the umbilical cord or used repeatedly. Another difficulty in comparing iodine formulations, aside from the application method, lies in the varying effective concentrations of iodine in different formulations (e.g., iodine vs. povidone-iodine vs. iodine tincture). Formulations in combination with alcohol (tinctures), for example, are said to enhance UCR drying due to the effect of alcohol [16], although this effect has not been demonstrated. Additionally, they are less effective against bacteria, requiring a longer contact time [15,16]. Besides different active substances (e.g., iodine or povidone-iodine), varying concentrations of the same substance are available (e.g., 5% or 10% povidone-iodine), which can lead to potential differences in effectiveness [60]. For example, the preparation that is recommended for administering into the UCR contains 10% povidone-iodine [25]. This high concentration compared to other iodine formulations (e.g., 5% povidone-iodine spray for multiple applications [26]) might impact the effectiveness of preventing omphalitis or even result in a higher risk of umbilical infections. A study on NC in foals showed a higher risk of intraabdominal infections when 2% PVP-iodine was applied once daily for 5 days compared to a shorter application time due to skin irritations caused by frequent application [60]. Potential skin irritations due to higher concentrations of PVP-iodine have already been described in infants [75]. In this study, however, no analyses of the respective preparation were possible due to the study design.

The odds of omphalitis tended to be higher on farms where farm workers wore gloves while performing NC compared to those who did not wear gloves. A similar tendency has already been shown by Ágredo-Campos et al. for the milking process. The incidence of *Staphylococcus aureus* was higher on farms that wore disposable gloves during their milking routine [76]. In general, however, gloves have been shown to reduce bacterial contamination and somatic cell count during the milking process [76,77]. However, it is not the wearing of the gloves themselves that is decisive, but the hygienic condition of the gloves. Workers may have a false sense of security and use contaminated gloves for NC due to handling calves, cows, or farm equipment immediately before NC, which can lead to contamination of the UCR. It is possible that contamination with faeces on the gloves may not be noticed. In contrast, contamination of the hands would be cleaned off immediately, leading to less contamination of the UCR. Unfortunately, no studies have been conducted on this presumption. Nevertheless, clean gloves (single use) should be worn to avoid the transmission of pathogens between farm workers and calves due to the transient flora on the worker’s hands (e.g., *Staphylococcus aureus*) [29,78]. It has been shown that the bacterial load can be reduced by 75% when wearing gloves compared to bare hands [79].

Regarding pediatrics literature, the recommendations to perform NC at all differ between birth settings. In hygienic community settings, most studies found no preventive effect of NC for the risk of omphalitis or mortality compared to dry cord care (no treatment), independent of the antiseptic used (e.g., CHX, alcohol in different concentrations) [31,32,35,36,80]. In most studies, even a prolonged time until UCR detachment was described after the use of NC [31,32,80]. In the case of resource-poor settings with high omphalitis incidences or after contamination of the UCR with excrements, however, NC is advised [33,65,71].

As hygienic circumstances on dairy farms are more comparable to those in resource-poor settings than in community settings, recommendations concerning NC in non-hygienic settings should be considered. Moreover, recommendations for dairy farmers based on the umbilical health status of their calves may be reasonable. Farms with less than 5% umbilical infections and hernias are recommended to focus on hygiene of the calving area, immediate separation of calf and dam, and proper colostrum management. Farms with problems in the umbilical health of their calves (> 5% umbilical infections and hernias), however, are recommended to additionally disinfect the navel repeatedly right after birth [45,68]. Nonetheless, on farms with hygienic, colostral, or management deficiencies, NC alone is insufficient to prevent umbilical infections. The hygienic circumstances on the farm, in general, play a much greater role in the development of omphalitis. Recent studies have shown that the hygiene of the calving pen and the calves’ housing are significant risk factors for umbilical health [4,12]. Thus, NC tends to reduce the bacterial load of the UCR [23] but may not be able to compensate for extensive hygienic deficiencies around birth.

Nonetheless, some limitations of the study design complicate a general recommendation towards or against NC. Goodness-of-fit statistics were carried out for all statistical models. Nevertheless, the models were not selected based on these results but on a hypotheses-based approach using causal diagrams.

Due to the study design, follow-up examinations to assess individual swellings of the umbilicus were not possible, which complicated a proper diagnosis of omphalitis. Due to the study’s definition of omphalitis, an enlarged umbilicus was classified as a case of omphalitis. Investigations in piglets showed that in the first three days of life, a proper diagnosis of omphalitis is not possible [81]. Unfortunately, no strict cut-off values or methods were implemented in our study to define enlargement as inflammation. Although training sessions for veterinarians were conducted to improve inter-observer agreement (but not intra-observer agreement) between veterinarians in the three study regions, misclassification of the umbilical status may still be possible due to the study definition. While some study veterinarians might have strictly adhered to the study definition and classified any enlargement as inflammation, other veterinarians may have categorised swellings in the first days of life as physiological. Therefore, false-positive cases were possible, leading to a potential bias in the results of the clinical examinations. Furthermore, neither the colostrum supply of each calf nor the hygienic status of the calving area at calving time could be evaluated. Farms experiencing problems in these areas and with umbilical health may tend to perform NC more frequently than farms with fewer problems. Therefore, a distortion of the effects of NC is possible. Moreover, only the management instruction for NC, not the actual implementation, was evaluated. The daily execution of NC may differ from the instructions of the farm manager due to variations in farm workers, individual calves, time of day, difficulties with the equipment used (e.g., unhygienic conditions), or incorrect implementation of NC. The deviation between farm instructions asked by a questionnaire and the actual implementation is a practical limitation of questionnaires [82]. Therefore, the executing person(s) should be accompanied and interviewed during their daily routine to evaluate the actual situation on the farms, including various NC practices and not just the antiseptic used.

## Conclusion

Considering the results of this study, performing navel care (NC) appears to be relevant in reducing the occurrence of omphalitis in neonatal dairy calves. Performing routine NC and applying preparations for NC repeatedly tended to reduce the odds of omphalitis. The preparations widely used for NC in neonatal calves include iodine compounds in various formulations, which are authorized for disinfection measures in food-producing animals. However, unwanted side effects in animals and users may occur. We expected that using iodine would influence the occurrence of omphalitis. However, none of the investigated preparation groups used for NC could reduce the odds of omphalitis compared to no NC. Administering the preparation into the umbilical cord, however, which was not very common (11% of farms, 13% of calves), could be determined to reduce the odds of omphalitis compared to no NC. This finding was unexpected due to the potential irritation of the intraabdominal tissue by iodine and the fact that farm workers need to touch the navel to administer the preparation into the umbilical cord, which may lead to further contamination and a higher risk of omphalitis. This fact and the observation that in Germany, different formulations of iodine (iodine vs. povidone-iodine vs. iodine tincture) in various concentrations (5% vs. 10%) with varying instructions for use (pouring on vs. spraying vs. administering into the UCR, and single vs. repeated application) are approved for NC, suggest that the different iodine preparations (especially the combination of formulation and concentration) might be the key to a successful NC strategy. Therefore, further well-controlled on-farm studies evaluating different NC practices and various iodine formulations on farms with differing umbilical health statuses are necessary to describe their impact on the occurrence of umbilical infections.

## Supporting information

S1 Fig Causal directed acyclic graph (DAG) (http://www.dagitty.net/) with “Ex_omphalitis” as target variable, “Q_practice_of_navel_care” as influence variable and all possible confounder variables. (PDF)

S2 Fig Causal directed acyclic graph (DAG) (http://www.dagitty.net/) with “Ex_omphalitis” as target variable, “Q_method_of_application_NC” as influence variable and all possible confounder variables. (PDF)

S3 Fig Causal directed acyclic graph (DAG) (http://www.dagitty.net/) with “Ex_omphalitis” as target variable, “Q_administering_preparation_into_umbilical_cord” as influence variable and all possible confounder variables. (PDF)

S4 Fig Causal directed acyclic graph (DAG) (http://www.dagitty.net/) with “Ex_omphalitis” as target variable, “Q_preparation_applied_for_NC” as influence variable and all possible confounder variables. (PDF)

S5 Fig Causal directed acyclic graph (DAG) (http://www.dagitty.net/) with “Ex_omphalitis” as target variable, “Q_frequency_of_NC” as influence variable and all possible confounder variables. (PDF)

S6 Fig Causal directed acyclic graph (DAG) (http://www.dagitty.net/) with “Ex_omphalitis” as target variable, “Q_time_of_first_NC” as influence variable and all possible confounder variables. (PDF)

S7 Fig Causal directed acyclic graph (DAG) (http://www.dagitty.net/) with “Ex_omphalitis” as target variable, “Q_wearing_gloves_during_NC” as influence variable and all possible confounder variables. (PDF)

S1 Table List of questions asked during the interview with the farm or herd manager that were included in this analysis. (PDF)

S2 Table List of hypotheses formulated prior to the analysis regarding the association of different practices of navel care (NC) and the occurrence of omphalitis in neonatal dairy calves. (PDF)
