# Supplementary material for: Field study on routine procedures for navel care in neonatal calves on dairy farms in Eastern Germany
Source: PLoS One. 2025 Jul 30;20(7):e0329326. doi: 10.1371/journal.pone.0329326 (PMC12309999; doi:10.1371/journal.pone.0329326)
Supplement: S7 Fig — (PDF) [file pone.0329326.s007.pdf]

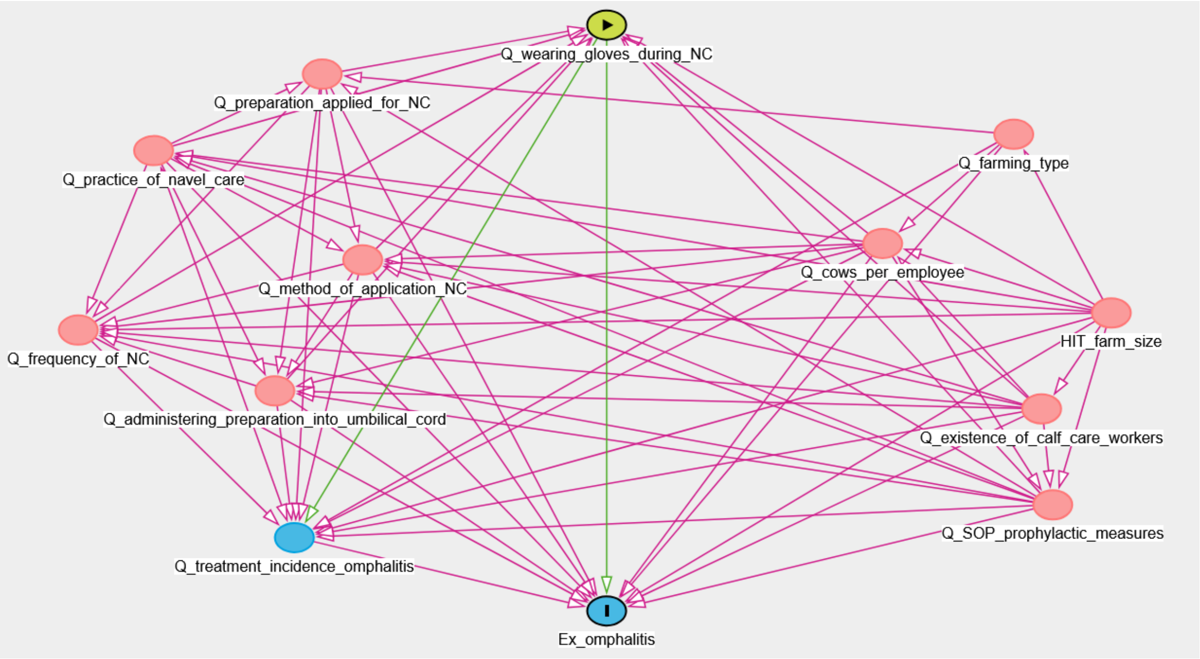

S7 Fig. Causal directed acyclic graph (DAG) (http://www.dagitty.net/) with "Ex_omphalitis" as target variable, "Q_wearing_gloves_during_NC" as influence variable and all possible confounder variables.

This Figure presents the causal directed acyclic graphs (DAG) (http://www.dagitty.net/) used to investigate the association between the use of gloves during navel care and the occurrence of omphalitis in neonatal dairy calves.

The arrows drawn demonstrate the connection between the variables included. The confounder variables (pink variables and arrows) were marked by the program (DAGitty) based on the arrows drawn.

Explanation of the abbreviation for each source the data was retrieved from:

Q: data retrieved by questionnaire

HIT: data retrieved from the National Traceability and Information System for Animals ("Herkunftssicherungs- und Informationssystem für Tiere", HIT)

Ex: data collected by clinical examination during a single farm visit

SOP: standard operation protocol
