# Supplementary material for: Field study on routine procedures for navel care in neonatal calves on dairy farms in Eastern Germany
Source: PLoS One. 2025 Jul 30;20(7):e0329326. doi: 10.1371/journal.pone.0329326 (PMC12309999; doi:10.1371/journal.pone.0329326)
Supplement: S1 Table — (PDF) [file pone.0329326.s008.pdf]

S1 Table: List of questions asked during the interview with the farm or herd manager that were included in this analysis.

Questions asked	Answer options
**Farm management**	
Is the farm run conventional or organic?	a) Conventional<br>b) Organic<br>c) In conversion to become organic<br>d) I don't know<br>e) No answer
Is there a person on this farm who is only responsible for the calves?	a) Yes<br>b) No<br>c) I don't know<br>d) No answer
How many people are employed on this dairy farm working full- or part-time? (Only people working with animals, no secretaries etc.)	Full-time: ____ persons<br>part-time: ____ persons
**Calving management**	
Where did most of the calvings take place in the 12 months preceding the farm visit?	Multiple answers were possible:<br>a) Stable (usual husbandry)<br>b) Calving pen (occupied by only one cow)<br>c) Calving Pen (occupied by multiple cows)<br>d) Combined pen for calving and diseased cows<br>e) Pasture<br>f) Other area<br>g) I don't know<br>h) No answer
In general, does the calf stay with the dam after birth for a specific time?	a) Yes<br>b) No<br>c) I don't know<br>d) No answer
Follow-up question: *If yes:* How many hours?	_____ hours
**Standard operation protocols (SOP)**	
Do standard operation protocols exist for health checks, prophylactic measures or treatments?	Multiple answers were possible:<br>a) Yes, for treatments<br>b) Yes, for health checks<br>c) Yes, for prophylactic measures<br>d) No<br>e) I don't know<br>f) No answer
Follow-up question: *If yes:* For which area?	Multiple answers were possible:<br>a) Calves

	b) Young Stock<br>c) Dry cows<br>d) Calving cows<br>e) Milking cows<br>f) I don't know<br>g) No answer
**Navel care**	
Is navel care performed?	a) Always (> 90% of the calvings)<br>b) Infrequently<br>c) Never<br>d) I don't know<br>e) No answer
Which preparation or product is applied for navel care?	a) Alcohol<br>b) Iodine-containing preparation<br>c) Chlorhexidine-containing preparation<br>d) Chlortetracycline spray<br>e) Other *(write down separately)*
How often is the umbilicus disinfected?	a) Once<br>b) Twice<br>c) Three times<br>d) More than 3 times<br>If more than once: How much time passes between the applications: ______ hours
How is the preparation applied?	a) Sprayed<br>b) Dipped<br>c) Poured-on<br>d) Painted/spotted
Is the preparation administered into the umbilical cord?	a) Yes<br>b) No
When is the umbilicus disinfected?	a) Immediately after birth<br>b) After approximately 6 hours<br>c) After approximately 12 hours<br>d) After approximately 24 hours<br>e) More than 24 hours
Are gloves worn while performing navel care?	a) Yes<br>b) No
