# Supplementary material for: Field study on routine procedures for navel care in neonatal calves on dairy farms in Eastern Germany
Source: PLoS One. 2025 Jul 30;20(7):e0329326. doi: 10.1371/journal.pone.0329326 (PMC12309999; doi:10.1371/journal.pone.0329326)
Supplement: S2 Table — (PDF) [file pone.0329326.s009.pdf]

S2 Table: List of hypotheses formulated prior to the analysis regarding the association of different practices of navel care (NC) and the occurrence of omphalitis in neonatal dairy calves.

Potential influence factor regarding navel care (NC)	Hypothesis
Practice of NC	If NC is performed after birth, the odds of omphalitis are lower compared to no NC.
Method of application	The occurrence of omphalitis depends on the method of application of the preparation used (comparison of dipping, spraying, pouring-on and painting/spotting).
Administering the preparation into the umbilical cord	If the preparation used is administered into the umbilical cord, the odds of omphalitis are higher (compared to alcohol, chlorhexidine or chlortetracycline spray).
Preparation or product applied	If an iodine-containing preparation is used for NC, the odds of omphalitis are lower compared to the use of other preparations.
Frequency of NC	The more frequently the umbilicus is disinfected after birth, the lower the odds of omphalitis.
Time of first NC	The earlier the umbilicus is disinfected after birth, the lower the odds of omphalitis.
Wearing gloves during NC	If gloves are worn while performing NC, the odds of omphalitis are reduced.
